# Comparison of Fecal MicroRNA Isolation Using Various Total RNA Isolation Kits

**DOI:** 10.3390/genes15040498

**Published:** 2024-04-16

**Authors:** Theresa Lederer, Noam M. Hipler, Cosima Thon, Juozas Kupcinskas, Alexander Link

**Affiliations:** 1 Department of Gastroenterology, Hepatology and Infectious Diseases, Otto-von-Guericke University, 39120 Magdeburg, Germany; 2 Department of Gastroenterology and Institute for Digestive Research, Lithuanian University of Health Sciences, 44307 Kaunas, Lithuania; juozas.kupcinskas@lsmuni.lt

**Keywords:** microRNA, fecal biomarker, total RNA, microRNA kits

## Abstract

Fecal specimens have long been regarded as promising sources for gastrointestinal cancer screening and have, thus, been extensively investigated in biomarker research. MicroRNAs (miRNAs) are small, non-coding RNA molecules involved in regulating various biological processes. They are commonly dysregulated during tumor development and exhibit differential expression in feces. To assess the preanalytical feasibility of fecal miRNA analysis, we systematically compared the performance of commonly used total RNA extraction methods. Fecal samples from healthy subjects were utilized for this evaluation. Various methods, including miRNeasy, Universal, Trizol, RNeasy, and mirVana kits, were employed to isolate total RNA. MiRNA expression analyses were conducted using TaqMan or SYBR Green qRT-PCR for a subset of miRNAs, with externally spiked-in cel-miR-39 used for normalization. Most methods demonstrated similar performance in terms of the total RNA concentration and purity. Externally spiked cel-miR-39 and endogenous miRNAs (RNU6b, miR-16, and miR-21) exhibited comparable concentrations across the different RNA isolation methods, whereas the RNeasy mini kit consistently yielded lower values. Our findings suggest that various isolation methods produce reproducible and comparable miRNA expression results, supporting the potential comparability and translational applicability of miRNA-based biomarker research in the future.

## 1. Introduction

Over the past few decades, biomarkers have revolutionized patient care and now play an essential role in the accurate diagnosis and management of virtually all diseases. According to Morrow and de Lemos, the assessment of the clinical potential of a novel biomarker may be structured into the following three domains: (1) reliable measurement, (2) consistent association between the biomarker and the disease, and (3) beneficial translational applications [1]. Although each of these points is crucial, the first point is particularly decisive in determining applicability during the initial stage. Reliable measurements encompass both pre- and analytical aspects, such as reproducibility, stability, and accessibility. To ensure reproducibility and comparability, standardized procedures based on the best possible processing methods must be integrated. However, in the context of cost-competitive medicine, factors such as time and cost also need to be taken into account to ensure the efficient use of the biomarker.

A non-invasive, sensitive, and specific screening tool is crucial for the early detection of various diseases, particularly cancer, such as CRC. Colonoscopy, while currently the gold standard for diagnosing inflammatory conditions and early neoplastic lesions, is invasive and costly. Additionally, a significant number of patients refuse colonoscopy [2], especially considering the changing cancer epidemiology with a rising incidence of early-onset CRC [3]. In this context, fecal samples have emerged as an excellent source for biomarker research. Although, historically, testing for fecal occult blood (FOBT) and the more advanced immunological FOBT (iFOBT) have been widely used, their diagnostic value is limited, highlighting the urgent need for improved molecular-based screening tools [4].

MicroRNAs (miRNAs) have emerged as new biomarkers for various diseases. MiRNAs are small, single-stranded RNA molecules that regulate the expression of certain proteins at the post-transcriptional level [5]. The miRNA has been detected in many different tissues and fluids, including ascites [6], blood [7], tumor tissue [8], bile [9,10], and feces [11,12]. It has been shown that, due to their biological regulatory function, they show altered expression patterns at an early stage, which is useful for diagnosis [13], and they can therefore be used to screen for malignant and chronic diseases such as chronic inflammatory bowel disease (IBD) [14,15] and hepatocellular carcinoma (HCC) [16] and also breast cancer [17]. In one of our pioneering studies [11], we were able to show that miRNA-21 and −106 were associated with an altered expression pattern in the stool of patients with colorectal cancer (CRC), and overall miRNAs in feces may have a unique diagnostic potential [18].

Besides CRC, other diseases may also benefit from the availability of diagnostic, prognostic, or predictive biomarkers, such as inflammatory bowel diseases (IBD). In this regard, others alongside us have explored the potential role of miRNA in the assessment of disease activity. Using the resting and validation cohort, we can show that miR-223 and miR-155, the inflammatory cells derived from miRNAs, show excellent diagnostic potential and correlate well with currently used fecal calprotectin in disease assessment [14]. Due to the increasing exploration of miRNA as a diagnostic tool, it is, however, crucial to delineate more specifically the preanalytical factor that may impact further exploration. Previously, it has been shown that there is substantial heterogeneity in the laboratory protocol, including the use of extraction kits, normalization methods, and applied evaluation tools in certain diseases to ensure applicability and reproducibility [19].

While several studies have been performed to investigate the performance of extraction methods for blood [13] or ascites samples [6], systematic analysis for fecal samples is still poorly understood. The aim of this study was to investigate the performance and comparability of commonly used or widely available miRNA extraction kits for miRNA analysis using a systematic approach.

## 2. Materials and Methods

### 2.1. Stool Samples from Healthy Subjects

**Figure 1:**
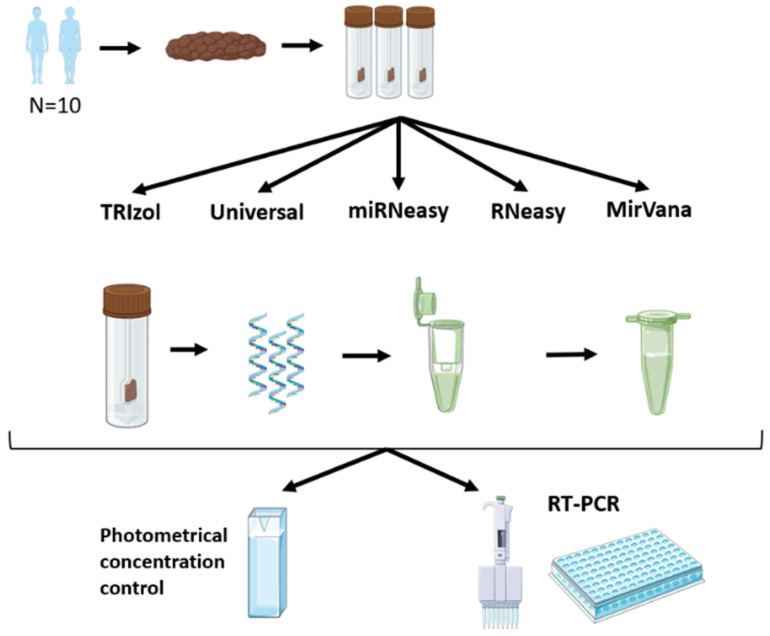
Graphical study design. Workflow of the study.

We collected 10 fresh stool samples from healthy individuals (5 for the main experiments and 5 for miRNeasy and mirVana comparison) for subsequent miRNA analysis. Following collection, a similar amount of approximately similar volume and weight of feces (50~100 µg) was aliquoted in separate vials for subsequent systematic extractions. The consistent weight distribution of the fecal samples was ensured using a standardized spatula for every sample. The fecal samples from each volunteer were homogenized, and the same amount of feces (100 µL each) was used for all extractions. The study protocol was approved by the institutional review board at Otto-von-Guericke University Magdeburg, Germany (99/10), and written informed consent was obtained from each volunteer. Stool samples were stored at −80 °C after collection, and subsequent RNA isolation was performed. Spiked in cel-miR-39 was added at a concentration of 5 ng/µL during the first step extraction for all extraction protocols for subsequent normalization (Figure 1).

### 2.2. RNA Extraction Using the TRIzol Method

Total RNA (including miRNAs) from fresh stool samples was extracted using the TRIzol method according to the manufacturer’s instructions, and minor modifications were applied (TRIzol, Invitrogen, Waltham, MA, USA). A similar amount of stool was homogenized with the TRIzol reagent, and the aqueous phase was collected in a new collection tube after centrifugation. This procedure was repeated 3 times. In the first round, 0.2 mL of chloroform was added; in the second wash, 0.5 mL of isopropanol was added; and in the third wash, 1 mL of 100% ethanol was added. The RNA pellet was then resuspended in RNase-free water. No silica-based membrane and no microspin technology were used for this extraction method (Figure 1).

### 2.3. RNA Extraction Using Qiagen Kits

Total RNA (including miRNAs) from fresh stool samples was extracted using QIAGEN miRNeasy Mini Kits, RNeasy Kits, and RNeasy Universal Plus Kits according to the manufacturer’s instructions with slight modifications (Qiagen, Hilden, Germany). Briefly, the aliquoted stool samples were homogenized with RNase-free water, and 100 μL of this homogenate was lysed at a ratio of 1:6 using the QIAzol lysis reagent (Qiagen, Valencia, CA). After homogenization, the RNA was precipitated with chloroform. The aqueous phase was mixed with a 1.5 volume of 100% ethanol and injected into the RNeasy Mini column. This column can be used to filter RNA from the sample according to the size of the RNA strand (Figure 1). One significant difference between the Qiagen kits is that the RNeasy kit does not use the phenol or QIAzol reagent, unlike the other two kits. For the RNeasy kit, 100% ethanol was used instead of 70% ethanol, and we did not use the enrichment step to select the small RNA molecules. No further modifications were made to the protocol.

### 2.4. RNA Extraction Using mirVana Kit

Total RNA (including miRNAs) from fresh stool samples was extracted using the mirVana kit according to the manufacturer’s instructions (mirVana, Invitrogen, Waltham, MA, USA). Aliquoted stool specimens were homogenized using the mirVana binding buffer, homogenate additive, and chloroform. After centrifugation, the aqueous phase was collected in a new collection tube. The aqueous phase was mixed with 100% ethanol and transferred to a silica-based membrane filter tube. The RNA was washed and filtered 3 times using different mirVana wash solutions.

### 2.5. MiRNA Quantification by Real-Time RT-PCR

The concentration of RNA extracted from stool samples was measured using RiboGreen RNA quantification kits (Molecular Probes, Eugene, OR, USA). The quantification of miRNA was performed using either TaqMan miRNA assays (Applied Biosystems Thermo Fisher Scientific, Waltham, MA, USA) or the SYBRgreen method. We analyzed externally spiked-in cel-miR-39 (Assay-ID: 0002000) and endogenous miRNA miR-16 (Assay ID: 000391), miR-21 and RNU6b [6,11,12]. Briefly, ~20 ng of RNA was reverse-transcribed, and real-time quantification was performed using an Applied Biosystems 7300 Sequence detection system. All reactions were performed in duplicates. Differences between groups are expressed as ΔCt (Figure 1).

### 2.6. Statistical Analysis

Data analyses were performed using Graph Pad Prism 9.5.1 software (San Diego, CA, USA). The differences between the two groups were analyzed using Student’s *t*-tests and between more than two groups using repeated measurements of ANOVA. The Benjamini-Hochberg method was used to determine the false discovery rate. Correlation analyses were performed using Pearson’s test. *p* values of <0.05 were regarded as significant.

## 3. Results

### 3.1. Total RNA Purity

**Figure 2:**
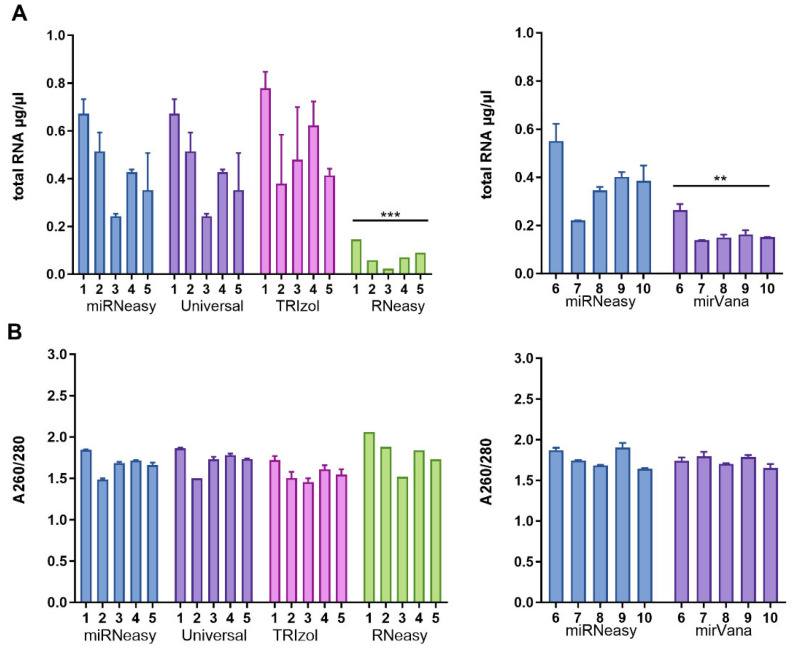
Total RNA and purity. (**A**) General comparison on total RNA level. (**B**) Comparison of purity of miRNAs. ** *p* ≤ 0.01; *** *p ≤* 0.001.

To explore the potential impact of extraction methods on the performance of miRNA expression analysis, we compared different extraction kits available on the market or previously used in fecal miRNA studies. To minimize the potential impact of disease-related factors, we specifically focused only on the samples from the healthy subjects (Figure 1). To obtain the first impression of the quality of the miRNAs and the extraction kits, the concentration of the isolated total RNA was compared in µg/µL (Figure 2A). The approximate concentration of the paired samples was ~0.3 to 0.9 µg/µL. There were no significant differences between the miRNeasy, Universal, and TRIzol kits. However, the RNeasy had a significantly lower total concentration compared to another test (*p* < 0.001), and mirVana was significantly lower compared to the miRNeasy kit in paired analysis (*p* = 0.0038). For the concentration of the miRNeasy and mirVana, analyses showed a total concentration of approximately 0.05 to 0.3 µg/µL.

In a second experiment, we compared the A260/A280 values following different extraction methods (Figure 2B). This index, with an ideal value of 1.8–2.0, represents the ratio of extracted nucleic acids with an absorption maximum of 260 nm and proteins with a maximum of 280 nm. In addition, this ratio can also be altered by contamination with, for example, phenol, which has been used. Relatively homogeneous results were obtained for all kits tested, concluding that all evaluated kits provided comparable results for the hard-to-use fecal specimens. Correlation analysis was performed to evaluate the possible correlation between the kits at different levels of comparison (Figure 2C)**.** The correlation analysis for total RNA concentration between the kits revealed a significantly strong correlation between RNeasy and Universal kits (*p* = 0.01, r = 0.958). The correlation for the A260/A280 index was significant between the Universal and miRNeasy kits (*p* = 0.003, r = 0.981).

**Figure 3:**
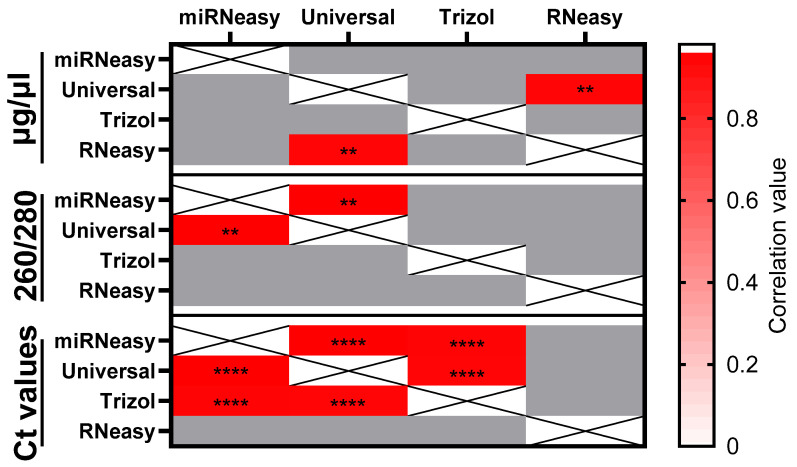
Correlation between the kits. Pearson correlation between the different kits based on Ct values and the amount of total RNA and RNA purity. ** *p* = 0.01; **** *p* = <0.0001.

At the level of Ct values of our extraction methods, a highly significant, very strong positive correlation was demonstrated between the miRNeasy kit and the TRIzol and Universal kits (miRNeasy/Universal *p* < 0.0001, r = 0.980; TRIzol/miRNeasy *p* < 0.0001 r = 0.959), and a highly significant, very strong positive correlation was found between the TRIzol and Universal kits (TRIzol/Universal *p* = <0.0001, r = 0.965) (Figure 3).

### 3.2. Comparison of miRNA Performance

**Figure 4:**
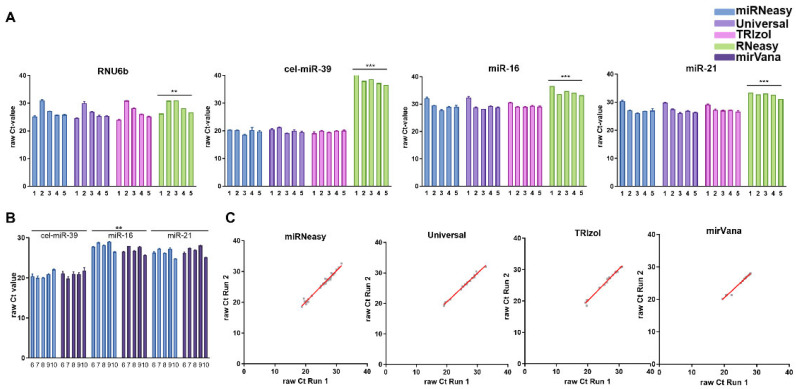
Performance of miRNA analysis and reproducibility analyses. (**A**) Data are presented as raw Ct for RNU6b and cel-miR-39, miR-16, and miR-21. (**B**) Data forming direct comparisons between miRNeasy and mirVana kits are presented as raw Ct for cel-miR-39, miR-16, and miR-21. (**C**) Extraction analyses for the independent qPCR run to confirm the validity of the data. ** *p ≤* 0.01; *** *p* ≤ 0.001.

To compare the performance of the different kits, we next compared the miRNA levels. A quantitative real-time PCR was performed for exogenous cel-miR-39, the commonly used exogenous normalizers miR-16 and RNU6b, as well as miR-21. First, we compared the raw Ct values to obtain an overview of the distribution of the qPCR results (Figure 4A). The analyses revealed a similar pattern, as shown for the total RNA analysis data. The raw CT values following the miRNeasy, Universal, and TRIzol kit extraction were comparable results without a significant difference for all studied miRNAs or snoRNA. The interindividual variation was higher for RNU6b and ranged from the Ct values 25 to 32. The lowest variation was, as expected for exogenous miRNA, observed for cel-miR-39. Overall, RNU6b showed higher raw Ct values (lower concentration) following RNeasy kit extraction, while the results among other extraction kits were comparable among extraction kits. Strikingly, the use of RNeasy extraction was associated with the substantial loss of exogenous cel-miR-39 (Ct range 35–40) in comparison to very similar results following extraction with other kits (*p* < 0.0001). In this experiment, the RNeasy kit also showed significantly different concentrations than the other kits. Figure 4B shows a similar comparison of the miRNeasy and mirVana kits. Here, the distribution shown in the total RNA analysis could not be confirmed, and no significant differences between the two kits were detected. The variation concentration pattern was comparable for the studied kits. Only for miR-16 was there a slightly higher miR-16 level following mirVana extraction (*p* = 0.01), although no significant difference was observed for other miRNAs.

We next aimed to confirm the reproducibility of the miRNA expression analysis. For this purpose, we performed an independent qPCR analysis of all miRNAs mentioned above, as shown in Figure 4C. The independent PCR analysis revealed for all tests a very high reproducibility miRNeasy kit *p* = 0.0001, r = 0.989; Universal kit *p* = 0.0001, r = 0.996; TRIzol-based extraction *p* = 0.0001; 0.995; mirVana kit *p* = 0.0001, r = 0.991.

### 3.3. Comparison of Normalized miR-21 Concentration

**Figure 5:**
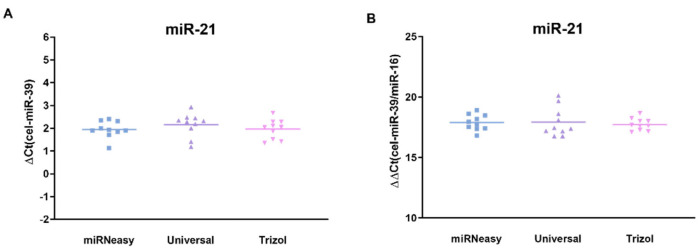
Comparison of normalized miR-21 levels. (**A**) miR-21 expression normalized to cel-miR-39 ΔCt method. (**B**) miR-21 expression normalized to combined cel-miR-39 and miR-16.

Finally, we questioned if using this systematic approach, there might be a potential difference among different kits in miR-21 levels following the normalization of PCR analysis. The normalization steps are usually required to evaluate the potential variation among subjects and a standard approach, although the question regarding the optimal normalization was not clarified for the fecal specimens. We applied two independent methods as follows: first, the exogenous spiked-in cel-miR-39 normalization (Figure 5A) and second, an additional normalization to miR-16 based on the previous suggestion that miR-16 might be considered as a normalizer (Figure 5B). The results of the analysis clearly show that evaluated extraction kits show comparable miR-21 levels following both normalization methods independent of kits.

## 4. Discussion

Fecal miRNA analysis has been introduced into the scientific pipeline for biomarker research for more than 10 years, but there are several issues related to the preanalytical evaluation that remain unanswered. In this study, we compared different miRNA extraction kits and their performance and comparability for fecal miRNA analysis using a systematic approach. For this purpose, we tested five different extraction kits for the quality and reproducibility of fecal miRNA results; we analyzed the purity of miRNA using the 260/280 ratio, followed by a systematic miRNA reproducibility analysis and a practical application to test the kits in a real-life setting. As a result, four out of five kits showed mostly homogeneous and comparable results, while one of the kits was associated with a significant analytical bias, suggesting its poor performance in translational research.

Before discussing the potential relevance of the data, it is essential to briefly outline the technical aspects and common steps associated with the evaluated extraction kits. Most of the methods, except the RNeasy extraction, included in the analysis were based on the acid-guanidinium thiocyanate–phenol–chloroform extraction method. In this method, three key chemicals play crucial roles in optimal performance. Guanidine thiocyanate acts as a highly water-soluble chaotropic agent, disrupting hydrogen bonding networks between molecules and denaturing proteins such as RNases, thereby separating rRNA from ribosomal proteins in the samples. Additionally, phenol dissolves proteins, while chloroform dissolves lipids. After centrifugation, the tube contains the following three distinct phases: the upper aqueous phase containing RNA, a middle phase containing DNA, and a lower phase containing proteins. The RNA in the aqueous phase can then be precipitated with isopropanol or ethanol [20,21,22]. For miRNA extraction, the use of an additional silica-based membrane filter tube is recommended to separate larger molecules (>200 nucleotides) from smaller ones [23,24]. Considering this particular aspect of the technical protocols, it is suggested that these kits may be comparable, albeit not identical to each other.

Numerous studies have compared various miRNA and RNA extraction methods across different body fluids and tissues, revealing significant diversity in their results [25,26,27]. For instance, Brunet-Vega et al. found that compared extraction kits yielded equivalent results for miRNA extraction from plasma in colorectal cancer patients [27], while Urbizu et al. achieved optimal extraction results using the mirVana kit for saliva samples [28]. Similarly, evaluations of miRNeasy, mirVana, and TRIzol kits for miRNA analysis in ascites demonstrated that miRNeasy and mirVana kits provided the most reliable results, whereas TRIzol-based extraction led to specific changes in miRNA ascites [6]. However, data regarding the performance of different extraction methods for fecal samples are lacking. Our study demonstrated that mirVana, miRNeasy, and the TRIzol method performed equally well with fecal samples, highlighting the importance of assessing each tissue type individually. Brunet-Vega et al. also suggested that the wide range of preanalytical factors affecting miRNA extraction in plasma may similarly apply to fecal samples [27]. Further research is needed to address potential confounding factors. New methods, such as magnetizable solid-phase extraction, as demonstrated by Bhati et al., show promise for DNA extraction and may also prove useful for miRNA isolation in the future [29]. Additionally, advancements in sequencing technologies and microarray techniques may offer alternative approaches, enabling the more accurate determination of miRNAs, albeit with evolving price-to-time ratios [30].

While interpreting our results, it is crucial to consider several key aspects. The observed similarity between the mirVana and miRNeasy kits in our study may be attributed to their similar membrane systems, which aligns with previous findings from our group comparing the extraction performance in ascites [6]. Interestingly, while the TRIzol methods introduced substantial bias compared to the aforementioned kits in previous studies, this difference was not observed in our analysis of fecal specimens. Our recent systematic analysis of different extraction kits in gastric cancer revealed the heterogeneous use of various methods for blood-based analysis [19]. Additionally, reports exist regarding the use of the RNeasy kit for miRNA extraction. Conversely, the markedly different results obtained with the RNeasy kit compared to other kits may also stem from differences in the membrane system and potentially the lack of phenol in the reactions. Notably, the manufacturer recommends using the miRNeasy kit instead of the RNeasy kit for miRNA extraction, as the filter systems of the latter are not optimized for miRNA isolation. There are no adapted instructions for using the RNeasy kit for small RNA molecules. The manufacturer suggests using a kit specified for miRNAs, but there are instructions for the Universal Plus kit, which can be used for both RNA and miRNA. This may be due to the use of phenol in the Universal Plus kit, which is missing in the RNeasy kit. Overall, the potential distinction between miRNeasy and RNeasy includes the use of 100% ethanol versus 70% ethanol. While this factor is frequently considered, there are still potential limitations in the use of the RNeasy extraction method. Based on our results, where we adapted the RNeasy protocol to 100% ethanol instead of 70% ethanol, there are still differences that do not allow an unbiased comparison between different studies, especially when spiked-in normalization for extraction is applied.

Having conducted research on miRNA over the past 15 years, we have frequently been asked about the potential and optimal extraction kits for translational miRNA research. Therefore, it is not surprising that we endeavored to adhere to a specific extraction kit to maximize reproducibility and comparability in our research while minimizing potential preanalytical factors that could affect the analysis. However, it is important to highlight several limitations associated with this systematic approach. First, our analysis was specifically conducted using a healthy population to minimize potential confounding effects related to disease states. Thus, we cannot exclude the possibility that in patients with inflammatory conditions, where the most significant data on the performance of fecal miRNA have been observed, there may be an impact of high blood-related factors on the performance of the extraction kits. Nonetheless, we believe that appropriate sample size reduction can mitigate this effect. Additionally, normalization using a spike in control is a widely used form of normalization in fecal samples [6,12,14]; nevertheless, normalization with endogenous normalizers is the best method of normalization, and this was lacking in our study; further endogenous normalizers in fecal samples are needed. Furthermore, we employed basic steps to compare the performance, but it would be intriguing to demonstrate the comparability for miRNA profiling using sequencing or array-based methods. One of the potential advantages that could be exploited in future studies is the use of the Bioanalyzer to gain more detailed insights into the efficiency of the extractions. However, this method may still have limitations when applied to the analysis of fecal samples. In particular, feces represent a mixture of RNA that includes not only miRNA but also, to a greater extent, bacterial and human RNA, which is often degraded and may significantly affect the validity of the results, although degraded mRNA is expected in fecal samples [31]. Therefore, in our experiments, we focused not only on RNA performance data but rather on the comparability of the miRNA level results, which we believe is the main determinant of the potential translational implementation of the method. Lastly, while our study employed a systematic approach using similar samples, it is conceivable that, with an increased sample size, differences may become more pronounced than demonstrated here.

In summary, despite more than a decade of exploration in biomarker research, fecal miRNA analysis still grapples with unresolved preanalytical challenges. Our study systematically evaluated five miRNA extraction kits, revealing homogeneous results for four kits but significant analytical bias in one, limiting its translational potential. Key technical aspects of extraction methods, notably the acid-guanidinium thiocyanate–phenol–chloroform approach, critically influence performance. Despite limitations, such as focusing on a healthy population and basic comparison steps, our findings emphasize the importance of a tailored assessment for each tissue type. Our results offer insights for researchers planning fecal miRNA-focused investigations and highlight the need for further exploration into optimal normalization and confounding factors, as well as the translational implications of miRNA in managing gastrointestinal diseases.

## Data Availability

The data presented in this study are available on request from the corresponding author since the authors have not received an agreement from participants for storage of the data in open domain.

## References

[1] Morrow D.A., De Lemos J.A. (2007). Benchmarks for the Assessment of Novel Cardiovascular Biomarkers. Circulation.

[2] Saengow U., Chongsuwiwatvong V., Geater A., Birch S. (2015). Preferences and Acceptance of Colorectal Cancer Screening in Thailand. Asian Pac. J. Cancer Prev..

[3] Patel S.G., Karlitz J.J., Yen T., Lieu C.H., Boland C.R. (2022). The Rising Tide of Early-Onset Colorectal Cancer: A Comprehensive Review of Epidemiology, Clinical Features, Biology, Risk Factors, Prevention, and Early Detection. Lancet Gastroenterol. Hepatol..

[4] Fitzpatrick-Lewis D., Ali M.U., Warren R., Kenny M., Sherifali D., Raina P. (2016). Screening for Colorectal Cancer: A Systematic Review and Meta-Analysis. Clin. Color. Cancer.

[5] Bartel D.P. (2018). Metazoan MicroRNAs. Cell.

[6] Schindler P., Kupcinskas J., Juzenas S., Skieceviciene J., Salteniene V., Schulz C., Weigt J., Malfertheiner P., Link A. (2018). Expression of MicroRNAs in the Ascites of Patients with Peritoneal Carcinomatosis and Peritonitis. Cancer Cytopathol..

[7] Pauley K.M., Satoh M., Chan A.L., Bubb M.R., Reeves W.H., Chan E.K. (2008). Upregulated MiR-146a Expression in Peripheral Blood Mononuclear Cells from Rheumatoid Arthritis Patients. Arthritis Res. Ther..

[8] Lu J., Getz G., Miska E.A., Alvarez-Saavedra E., Lamb J., Peck D., Sweet-Cordero A., Ebert B.L., Mak R.H., Ferrando A.A. (2005). MicroRNA Expression Profiles Classify Human Cancers. Nature.

[9] Li L., Piontek K.B., Kumbhari V., Ishida M., Selaru F.M. (2016). Isolation and Profiling of MicroRNA-Containing Exosomes from Human Bile. J. Vis. Exp..

[10] Li L., Masica D., Ishida M., Tomuleasa C., Umegaki S., Kalloo A.N., Georgiades C., Singh V.K., Khashab M., Amateau S. (2014). Human Bile Contains MicroRNA-Laden Extracellular Vesicles That Can Be Used for Cholangiocarcinoma Diagnosis. Hepatology.

[11] Link A., Balaguer F., Shen Y., Nagasaka T., Lozano J.J., Boland C.R., Goel A. (2010). Fecal MicroRNAs as Novel Biomarkers for Colon Cancer Screening. Cancer Epidemiol. Biomark. Prev..

[12] Link J., Thon C., Petkevicius V., Steponaitiene R., Malfertheiner P., Kupcinskas J., Link A. (2023). The Translational Impact of Plant-Derived Xeno-MiRNA MiR-168 in Gastrointestinal Cancers and Preneoplastic Conditions. Diagnostics.

[13] Guo Y., Vickers K., Xiong Y., Zhao S., Sheng Q., Zhang P., Zhou W., Flynn C.R. (2017). Comprehensive Evaluation of Extracellular Small RNA Isolation Methods from Serum in High Throughput Sequencing. BMC Genom..

[14] Schönauen K., Le N., von Arnim U., Schulz C., Malfertheiner P., Link A. (2018). Circulating and Fecal MicroRNAs as Biomarkers for Inflammatory Bowel Diseases. Inflamm. Bowel Dis..

[15] Zhou R., Qiu P., Wang H., Yang H., Yang X., Ye M., Wang F., Zhao Q. (2021). Identification of MicroRNA-16-5p and MicroRNA-21-5p in Feces as Potential Noninvasive Biomarkers for Inflammatory Bowel Disease. Aging.

[16] Sorop A., Constantinescu D., Cojocaru F., Dinischiotu A., Cucu D., Dima S.O. (2021). Exosomal MicroRNAs as Biomarkers and Therapeutic Targets for Hepatocellular Carcinoma. Int. J. Mol. Sci..

[17] Chiorino G., Petracci E., Sehovic E., Gregnanin I., Camussi E., Mello-Grand M., Ostano P., Riggi E., Vergini V., Russo A. (2023). Plasma MicroRNA Ratios Associated with Breast Cancer Detection in a Nested Case–Control Study from a Mammography Screening Cohort. Sci. Rep..

[18] Wang C., Zhao K., Rong Q. (2015). Diagnostic Value of Fecal MicroRNAs for Colorectal Cancer: A Meta-Analysis. Clin. Lab..

[19] Link A., Kupcinskas J. (2018). MicroRNAs as Non-Invasive Diagnostic Biomarkers for Gastric Cancer: Current Insights and Future Perspectives. World J. Gastroenterol..

[20] Chomczynski P., Sacchi N. (2006). The Single-Step Method of RNA Isolation by Acid Guanidinium Thiocyanate–Phenol–Chloroform Extraction: Twenty-Something Years On. Nat. Protoc..

[21] Chomczynski P., Sacchi N. (1987). Single-Step Method of RNA Isolation by Acid Guanidinium Thiocyanate-Phenol-Chloroform Extraction. Anal. Biochem..

[22] Perry R.P., La Torre J., Kelley D.E., Greenberg J.R. (1972). On the Lability of Poly(A) Sequences during Extraction of Messenger RNA from Polyribosomes. Biochim. Biophys. Acta BBA Nucleic Acids Protein Synth..

[23] McCormick R.M. (1989). A Solid-Phase Extraction Procedure for DNA Purification. Anal. Biochem..

[24] Tan S.C., Yiap B.C. (2009). DNA, RNA, and Protein Extraction: The Past and The Present. J. Biomed. Biotechnol..

[25] Wright K., de Silva K., Purdie A.C., Plain K.M. (2020). Comparison of Methods for MiRNA Isolation and Quantification from Ovine Plasma. Sci. Rep..

[26] Ding M., Wang C., Lu X., Zhang C., Zhou Z., Chen X., Zhang C.-Y., Zen K., Zhang C. (2018). Comparison of Commercial Exosome Isolation Kits for Circulating Exosomal MicroRNA Profiling. Anal. Bioanal. Chem..

[27] Brunet-Vega A., Pericay C., Quílez M.E., Ramírez-Lázaro M.J., Calvet X., Lario S. (2015). Variability in MicroRNA Recovery from Plasma: Comparison of Five Commercial Kits. Anal. Biochem..

[28] Urbizu A., Arnaldo L., Beyer K. (2023). Obtaining MiRNA from Saliva—Comparison of Sampling and Purification Methods. Int. J. Mol. Sci..

[29] Bhati A., Varghese A., Rajan G., Sridhar V., Mohan Y., Pradeep S., Babu S., Kaikkolante N., Sarma M., Arun S. (2021). An Effective Method for Saliva Stabilization and Magnetic Nanoparticles Based DNA Extraction for Genomic Applications. Anal. Biochem..

[30] Pritchard C.C., Cheng H.H., Tewari M. (2012). MicroRNA Profiling: Approaches and Considerations. Nat. Rev. Genet..

[31] Link J., Thon C., Schanze D., Steponaitiene R., Kupcinskas J., Zenker M., Canbay A., Malfertheiner P., Link A. (2019). Food-Derived Xeno-MicroRNAs: Influence of Diet and Detectability in Gastrointestinal Tract—Proof-of-Principle Study. Mol. Nutr. Food Res..

